# Enrichment of small pathogenic deletions at chromosome 9p24.3 and 9q34.3 involving *DOCK8, KANK1, EHMT1* genes identified by using high-resolution oligonucleotide-single nucleotide polymorphism array analysis

**DOI:** 10.1186/s13039-016-0291-3

**Published:** 2016-11-15

**Authors:** Jia-Chi Wang, Loretta W. Mahon, Leslie P. Ross, Arturo Anguiano, Renius Owen, Fatih Z. Boyar

**Affiliations:** Cytogenetics Laboratory, Quest Diagnostics Nichols Institute, 33608 Ortega Highway, San Juan Capistrano, CA 92690 USA

**Keywords:** Small pathogenic deletions, High resolution oligonucleotide-single nucleotide polymorphism array analysis, Haploinsufficiency, Homozygous deletions

## Abstract

**Background:**

High-resolution oligo-SNP array allowed the identification of extremely small pathogenic deletions at numerous clinically relevant regions. In our clinical practice, we found that small pathogenic deletions were frequently encountered at chromosome 9p and 9q terminal regions.

**Results:**

A review of 531 cases with reportable copy number changes on chromosome 9 revealed142 pathogenic copy number variants (CNVs): 104 losses, 31 gains, 7 complex chromosomal rearrangements. Of 104 pathogenic losses, 57 were less than 1 Mb in size, enriched at 9p24.3 and 9q34.3 regions, involving the *DOCK8, KANK1, EHMT1* genes. The remaining 47 cases were due to interstitial or terminal deletions larger than 1 Mb or unbalanced translocations. The small pathogenic deletions of *DOCK8, KANK1* and *EHMT1* genes were more prevalent than small pathogenic deletions of *NRXN1, DMD, SHANK3* genes and were only second to the 16p11.2 deletion syndrome, 593-kb (OMIM #611913).

**Conclusions:**

This study corroborated comprehensive genotype-phenotype large scale studies at 9p24.3 and 9q24.3 regions for a better understanding of the pathogenicity caused by haploinsufficiency of the *DOCK8, KANK1 *and *EHMT1* genes.

**Trial registration number:**

None; it is not a clinical trial, and the cases were retrospectively collected and analyzed.

**Electronic supplementary material:**

The online version of this article (doi:10.1186/s13039-016-0291-3) contains supplementary material, which is available to authorized users.

## Background

Chromosomal microarray analysis (CMA) has been widely utilized for the genome-wide screening of microdeletion and microduplication syndromes [1]. The sizes of well-known microdeletion and microduplication syndromes were usually larger than 1 Mb, such as 1.4 Mb for Williams-Beuren syndrome (OMIM #194050) or 2.8 Mb for DiGeorge syndrome (OMIM #188400). Small (<1 Mb) pathogenic deletions at regions which were not well characterized were frequently encountered during our daily clinical practice, for instance, the chromosomal regions at 9p24.3 and 9q34.3.

High-resolution oligo-SNP array is able to reveal a variety of chromosomal disorders including uniparental disomy or extremely small pathogenic deletions which would be missed by low-resolution oligonucleotide CMA. Our and other previous studies showed the cases with uniparental disomy were relatively limited in number on chromosome 9 as compared to chromosome 15, 11 and 7 [2, 3]. In contrast, small pathogenic deletions were frequently encountered at chromosome 9p24.3 and 9q34.3 by using high-resolution oligo-SNP array in postnatal studies. Research endeavors have been significantly prioritized to specific genes such as *NRXN1* and *SHANK3* in the past [4, 5]. To the best of our knowledge, only four cases with small deletions of 192 kb, 225 kb, 465 kb and 518 kb in size at 9p24.3 involving the *DOCK8* and/or *KANK1* gene [6–8], and a case of 40 kb deletion in the *EHMT1* gene at 9q34.3 [9] have been documented.

The purpose of this study is to evaluate 1): the incidence of small (< 1 Mb) pathogenic deletions in postnatal specimens, 2): whether the small pathogenic deletions at 9p24.3 and 9q34.3 constituted a significant proportion of small deletions, 3): what proportion of deletions on chromosome 9 was caused by small pathogenic deletions at 9p24.3 and 9q34.3, 4): the efficacy of identifying extremely small homozygous pathogenic deletions using high-resolution oligo-SNP array.

## Results

### The incidence of small pathogenic deletions in postnatal specimens studied by high-resolution oligo-SNP array

**Fig. 1:**
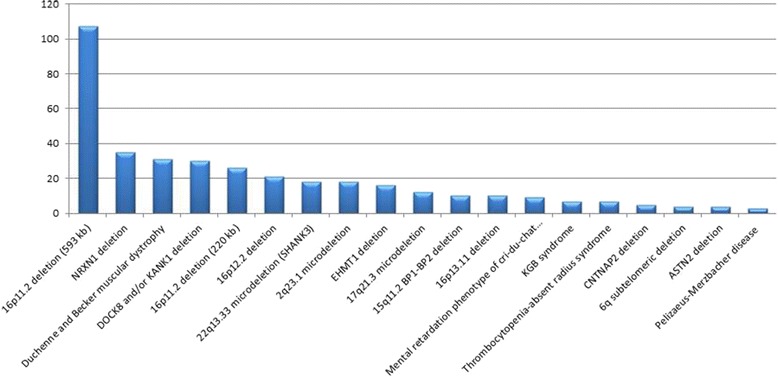
The recurrent small pathogenic small copy number losses from all the chromosomes. The small pathogenic deletions on chromosome 9, involving *DOCK8, KANK1, EHMT1* genes constituted a significant proportion (12 %) of cases

Approximately 38,000 postnatal specimens were studied by high-resolution oligo-SNP array in our laboratory from 2011 through 2015. Of these, we reported approximately 13,000 (34 %) pathogenic variants or variants of uncertain clinical significance (VOUS). The detection rate was consistent with our previous study [2]. Of the 13,000 variants, a total of 373 recurrent (at least 3 cases) small pathogenic losses were identified (Fig. 1). The 16p11.2 deletion syndrome, 593-kb (OMIM #611913) is the most common small pathogenic loss (107 cases). The remaining involved *NRXN1* (35 cases), *DMD* (31 cases), *DOCK8* and/or *KANK1* (30 cases), and other chromosomal regions such as 16p11.2 (OMIM #613444, 220-KB), 16p12.2 (OMIM #136570, 520-KB), 22q13.33 (OMIM #606232).

### Approximately 12 % of the recurrent small pathogenic copy number losses were caused by deletions of *DOCK8 andKANK1* at 9p24.3 and *EHMT1* at 9q34.3

**Fig. 2:**
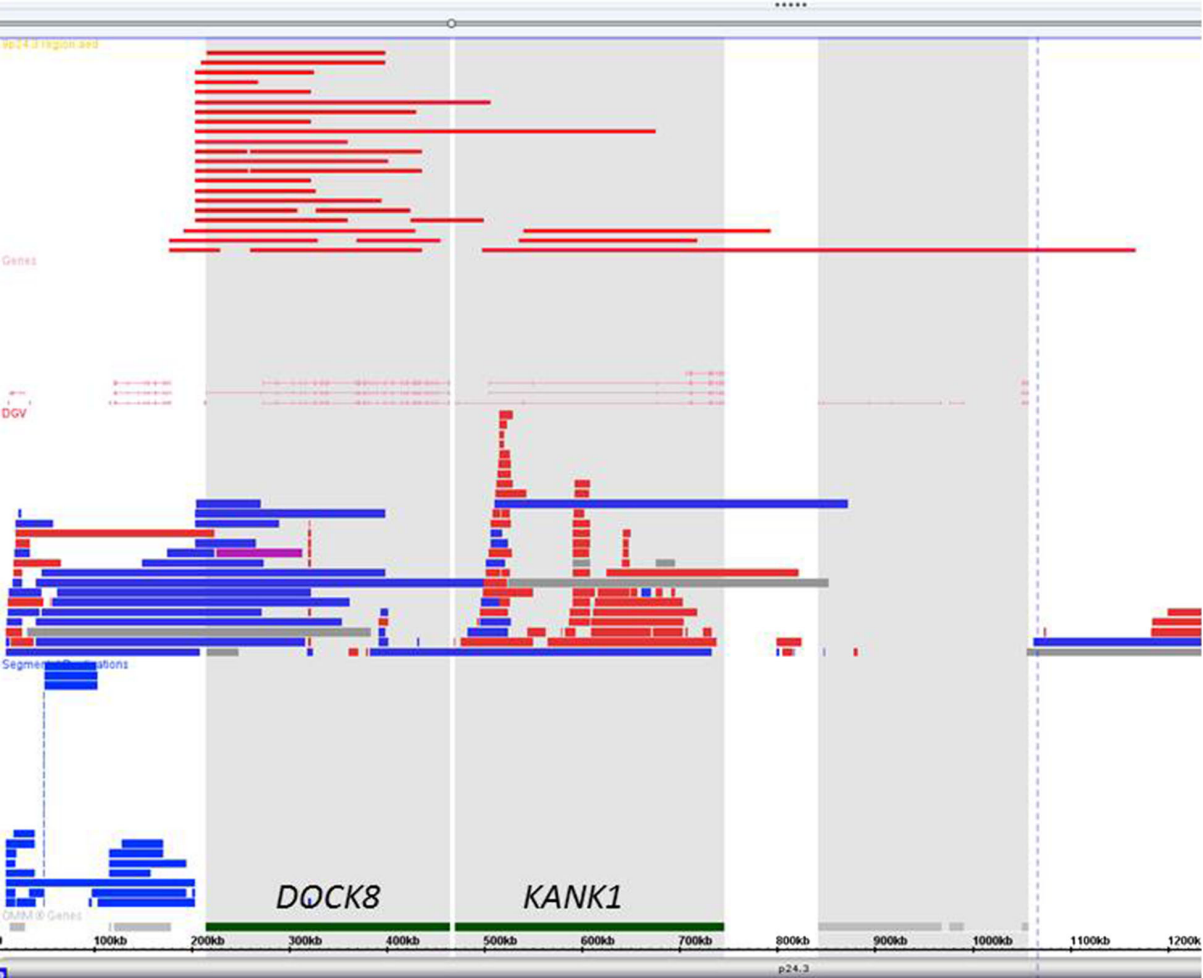
The distribution of deletions in cases with involvement of the *DOCK8* and *KANK1* genes. The copy number variants in the database of genomic variation were compared to the profile ﻿of﻿ small deletions in our cohort. Segmental duplications were not flanking the deletions of the *DOCK8* and *KANK1 *genes, which potentially excluded the pathogenic mechanism through non-homologous recombination of segmental duplications

**Fig. 3:**
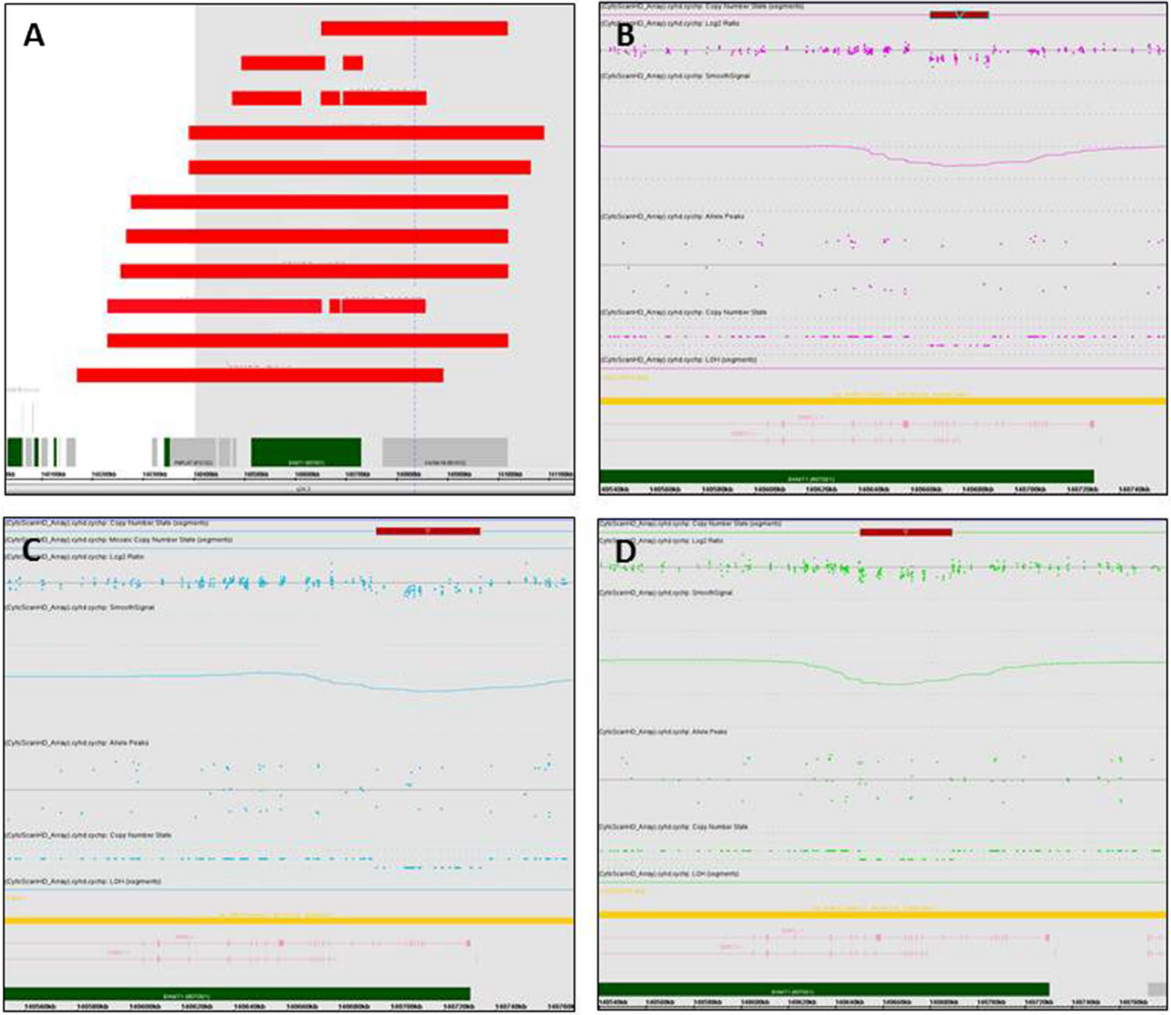
**a** The genomic locations of the 16 small (<1 Mb) pathogenic deletions involving the *EHMT1* gene. **b**. A 22-kb deletion (chr9:140,667,619-140,689,934) was found in 32-year-old female with intellectual disability. **c** A 40-kb deletion (chr9:140,694,541-140,734,178) was identified in a 5-year-old girl. **d** A 39-kb deletion (chr9:140,650,471-140,689,373) was discovered in a 1-year-old girl with developmental delay, speech and motor delay, white matter changes on MRI, and hypotonia

The total number of deletions involving *DOCK8* and/or *KANK1* genes at 9p24.3 (30 cases, Fig. 2) and *EHMT1* gene at 9q34.3 (16 cases, Fig. 3a) constituted approximately 12 % (46/373 cases) of the recurrent small pathogenic deletions from all the chromosomes.

### Small (<1 Mb) and relatively small (1–1.5 Mb) deletions constituted 59 % of pathogenic copy number losses on chromosome 9

Of 13,000 cases with reported copy number variants (CNVs), 531 cases were from chromosome 9, and 142 were pathogenic, including 104 losses, 31 gains, 7 complex chromosomal rearrangements with both losses and gains (Table 1 and Additional file 1: Figure S1). Of 104 pathogenic losses, 57 were smaller than 1 Mb in size. Of 57 cases, 46 were located at 9p24.3 and 9q34.3 regions, involving the *DOCK8, KANK1, EHMT1* genes. The size ranged from 22 kb (case 54 with *EHMT1* deletion) to 790 kb (case 35), with an average of 384 kb (Table 1 and Additional file 2: Table S1). The remaining 47 cases were either resulted from interstitial deletions over 1 Mb in size (18 cases), terminal deletions over 1 Mb (14 cases), or unbalanced translocations (15 cases) that could be as small as 880 kb in size in a t(9;11)(q34.3;p15.4)(case 47, Additional file 2: Table S2). Very interestingly, of these 47 cases, four had relatively small deletions (1–1.5 Mb in size): a 1,048-kb deletion (case 10, Additional file 2: Table S2) involving the *PTCH1* gene (OMIM 601309) and diagnosis of Gorlin syndrome (OMIM #109400), a 1,349-kb deletion (case 15) involving the *STXBP1* gene and diagnosis of early infantile epileptic encephalopathy (OMIM #612164), and two 9q34.3 deletions of 1,218 kb and 1,268 kb (case 31 and 32). The small (57 cases) and relative small (4 cases) deletions established 59 % (61/104) of pathogenic copy number losses on chromosome 9.

**Table 1:** Profile of pathogenic copy number losses and gains in chromosome 9

Type or gene involved	Number	Mean, kb	Range, kb
Copy number losses : large			
Interstitial deletion	18	5,070	1,048–16,837
Terminal deletion	14	5,273	1,218–13,593
Unbalanced translocation	15	7,053	880–17,107
Subtotal	47		
Copy number losses: small			
*DOCK8*	24	299	97–431
*KANK1*	3	369	184–670
*DOCK8* and *KANK1*	3	419	75–676
*EHMT1*	16	417	22–790
*ASTN2*	4	150	78–271
*FREM1*	2	369	166–573
*STXBP1*	1	592	NA
*COL5A1*	1	370	NA
*GLDC*	1	50	25–75
*TOPORS*	1	337	NA
*CDK5RAP2*	1	74	NA
Subtotal	57		
Large copy number gains			
Trisomy 9p/proximal 9q	15	45,996	13,826–95,453
9q duplications	6	12,172	6,277–18,399
Unbalanced translocation	4	25,717	6,794–68,089
Tetrasomy 9q and proximal 9q due to isochromosome	3	66,477	49,843–81,113
Trisomy 9	2	140,833	NA
Triplication	1	15,972	NA
Subtotal	31		
Complex chromosomal rearrangements (CCRs)			
Inverted duplication with terminal deletion of 9p	3	Deletion: 6,377; Duplication: 32,271	
Inverted duplication with terminal deletion of 9q	1	Deletion: 161; Duplication: 3,127	
Multiple deletion and duplication	2	Losses: 1091; Gains: 12,517	
Chromothripsis	1	Loss: 134; Gains: 21,094	
Subtotal	7		
Total	142		

*NA* not applicable

### The 9p24.3 (0–2.2 Mb from 9p telomere) and 9q34.3 (137.4–141.2 Mb from 9p telomere) were two hot spots of pathogenic copy number losses on chromosome 9

The 9p24.3 and 9q34.3 regions were two hot spots of copy number losses on chromosome 9. A total of 35 cases majorly involved the 9p24.3 region: 30 small pathogenic deletions (case 1–30, Additional file 2: Table S1), one interstitial deletion (case 1, Additional file 2: Table S2), two terminal deletions (case 19 and 20), and two unbalanced translocations (case 33 and 35). Additionally, 26 cases were localized to 9q34.3 region: 17 small pathogenic deletions (case 41–57, Additional file 2: Table S1), one interstitial deletion (case 18, Additional file 2: Table S2), 5 large terminal deletions (case 28–32) and 3 unbalanced translocations (case 45–47). Overall, close to 60 % (61/104, 59 %) of deletions were localized to the very ends of chromosome 9 at band 9p24.3 and 9q34.3.

### Rarely encountered extremely small homozygous pathogenic deletions were discovered in two cases

**Fig. 4:**
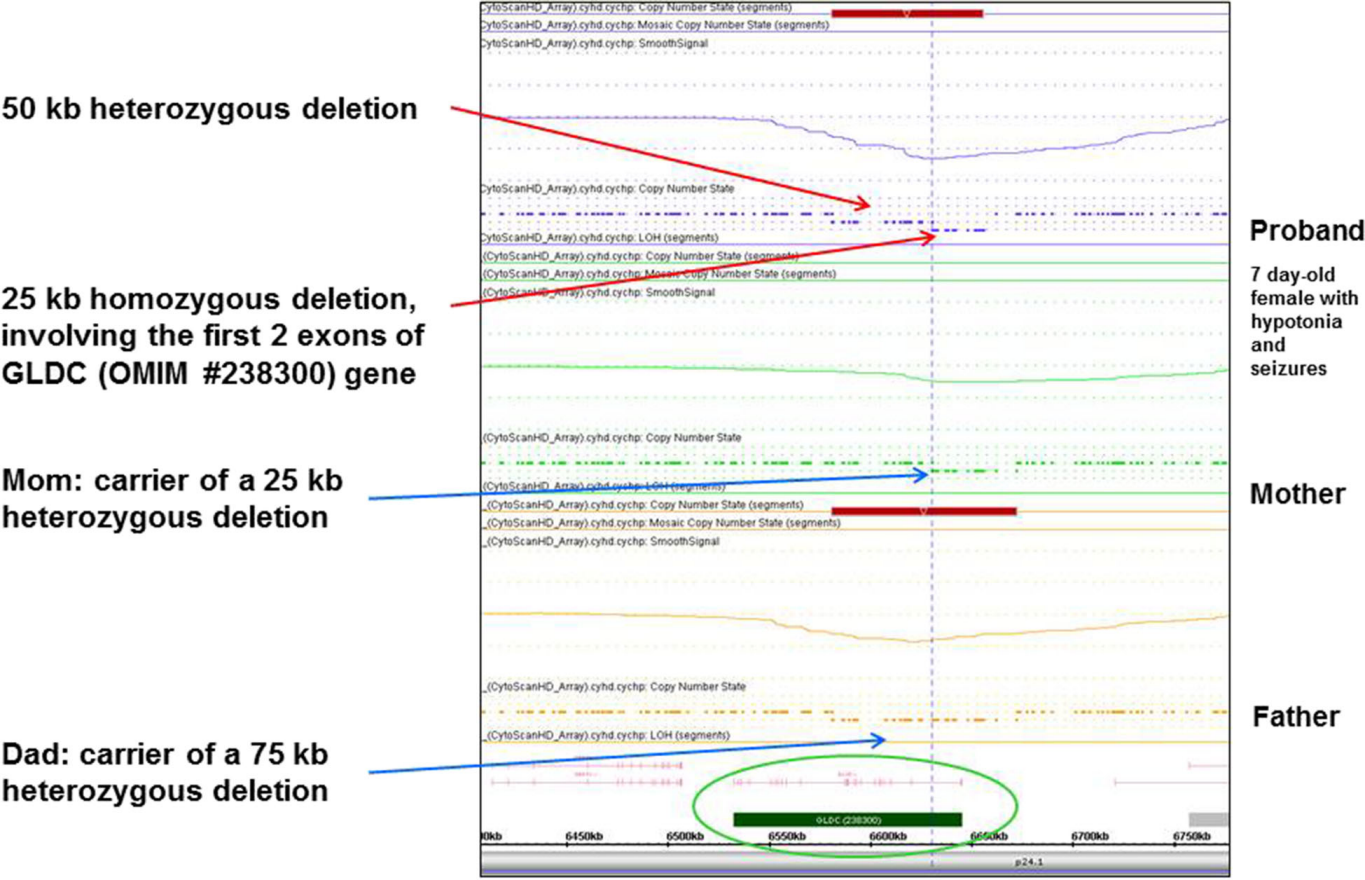
Family study of homozygous and heterozygous deletion of the *GLDC* gene. High-resolution oligo-SNP array analysis of the proband revealed a 25-kb homozygous and a 50-kb heterozygous deletion at the 5′ region of the *GLDC* gene. These two deletions involved multiple exons and led to autosomal recessive glycine encephalopathy (nonketotic hyperglycinemia; OMIM #605899). Family study showed the mother was a carrier of a 25-kb heterozygous deletion and the father was a carrier of a 75-kb heterozygous deletion of the *GLDC* gene. The 25-kb maternally inherited deletion was located within the 75-kb paternally inherited deletion, and thus led to a 25-kb homozygous and a 50-kb heterozygous deletion in the proband

Extremely small homozygous pathogenic deletions were identified in two cases: 1): a new born baby girl who presented with a metabolic disorder (abnormal reflexes, hypotonia, seizures, and elevated glycine) was revealed to contain a 25-kb homozygous deletion in the *GLDC* gene, which gave rise to autosomal recessive glycine encephalopathy (nonketotic hyperglycinemia; OMIM #605899). Besides that, a 50-kb heterozygous deletion was also found in the 5′ region of the *GLDC* gene. Parental study showed the mother was a carrier of a 25-kb heterozygous deletion and the father was a carrier of a 75-kb heterozygous deletion of the *GLDC* gene. The 25-kb maternally inherited deletion was located within the 75-kb paternally inherited deletion, and therefore inheritance of abnormal allele from both parents led to a 25-kb homozygous and a 50-kb heterozygous deletion in the proband (Fig. 4); 2): a 1-year-old boy was found to have a 74-kb homozygous deletion of the *CDK5RAP2* gene in a region of homozygosity (Additional file 3: Figure S2) which led to autosomal recessive primary microcephaly-3 (OMIM #604804). The presence of multiple large regions of homozygosity (a total of 421 Mb) implied these two parents were closely related in blood. The proband inherited the heterozygous abnormal allele with deletion of the *CDK5RAP2* gene from both parents.

## Discussion

The subtelomeric region such as 1p36 is known to be gene-rich and prone to have deletions, supported by a study with a large cohort of over 5,000 cases [10]. The cases with subtelomeric rearrangements comprised of about 46 % of all the genomic abnormalities identified by CMA [10]. However, as compared to 1p36, 22q13, 4p16, 5p15, very limited cases at the ends of chromosome 9p and 9q were established [10]. When we reviewed the profile of copy number losses from our database of 38,000 postnatal cases studied by using high-resolution oligo-SNP array and sorted it based on chromosomal regions, we discovered that all the cases with 1p36 deletions were over 1 Mb: 20 cases were 1–3 Mb, eight cases were 3–5 Mb, eight cases were 5–10 Mb and five cases were 10–20 Mb in size (unpublished data). In contrast, 61 of 104 pathogenic deletions on chromosome 9 were either smaller than 1 Mb (57 cases) or between 1 and 1.5 Mb (4 cases). This finding demonstrated that the size of copy number losses conspicuously varied between chromosomal regions. In the clinical practice, the concept of vigilant selecting appropriate methods to characterize the genomic losses at different regions becomes essentially important. For instance, FISH analysis using subtelomeric or locus-specific probe may ﻿be approriate to identify 1p36 microdeletions﻿, but may﻿ miss cases with small deletions on chromosome 9.

Extremely small intragenic deletion of the *EHMT1* gene was only reported in one case: a 40-kb intragenic deletion of the *EHMT1* gene with uncertain phenotypic consequence [9]. A more recent update on Kleefstra syndrome exhibited that the 16 newly diagnosed 9q34.3 deletions were all larger than 200 kb [11]. In our cohort, we identified a total of 24 cases with 9q34.3 deletions: 16 with small (<1 Mb) deletions involving the *EHMT1* gene (case 42–57, Additional file 2: Table S1), 5 with terminal deletion of 9q (case 28–32, Additional file 2: Table S2), and 3 with small (880–1001 kb in size) 9q34.3 deletions due to unbalanced translocations (case 45–47, Additional file 2: Table S2). Remarkably, we brought about three extremely small (22 kb, 39 kb and 40 kb in size) intragenic deletions of the *EHMT1* gene and all were clustered at the 3′ end of the gene (Fig. 3, b-d). The 22-kb deletion (Fig. 3b) was identified in a 32-year-old female with intellectual disability; whereas, the 40-kb deletion (Fig. 3c) was found in a 5-year-old girl and the 39-kb deletion (Fig. 3d) was discovered in a 1-year-old girl with typical features of 9q34.3 deletion including developmental delay, speech and motor delay, and hypotonia [9]. In addition, a 26-year-old male with a 165-kb deletion involving *EHMT1* and CACNA1B gene (case 57, Additional file 2: Table S1) also presented clinical features which were typical for 9q34.3 deletion syndromes including mental retardation, developmental delay, speech delay, motor delay, learning disability, autism spectrum disorder, asymmetry of temporal lobe, localized polymicrogyria, loping gait and scoliosis [11].

In contrast to the *EHMT1* gene of which haploinsufficiency score was much better established by ClinGen (https://www.ncbi.nlm.nih.gov/projects/dbvar/clingen/), the sensitivity to haploinsufficiency for *DOCK8* and *KANK1* genes was not proven (Additional file 2: Table S3). There were two unrelated patients with mental retardation and developmental disability (MRD2; OMIM #614113) who were disclosed to have a heterozygous disruption of the longest isoform of the *DOCK8* gene by either deletion or a translocation of t(X;9) [12]. A recent report of another two patients with almost identical deletion involving both *DOCK8* and *KANK1* displayed two distinct phenotypes [6]. The study of a four-generation with a 225-kb deletion of the *KANK1* gene implied that an imprinting mechanism may play a role in the phenotypic variation in this family. The authors suggested *KANK1* is a maternally imprinted gene and only expressed in the paternal allele [8]. However, other report did not support this finding [7]. In our cohort, deletions of *KANK1* were found in 6 cases (case 25–30, Additional file 2: Table S1). There were not enough clinical data to determine whether *KANK1* is a maternally imprinted gene. On the other hand, *DOCK8* gene is unlikely to be maternally imprinted since the two half-brothers (a 2-year-old boy and a 7-year-old boy, case 13 and 14, Additional file 2: Table S1) both inherited the deleted *DOCK8* allele from the same mother. In addition, our patients with small deletions of the *DOCK8* gene had very strong family history (case 13/14, 17, 20/21 Additional file 2: Table S1) and shared similar clinical features, including developmental delay and intellectual disability (4 out of 5 cases), speech and motor delay (3), learning disability (2), behavior problems or autism (3), macrocephaly (2), dysmorphic or congenital anomalies (4). Our cohort provided additional pathogenic ev﻿idence for haploins﻿ufficiency of *DOCK8* gene.

Although extremely rare, two cases with homozygous deletions of *GLDC* and *CDK5RAP2* genes were discovered in this cohort. In our previous study, we demonstrated the autosomal recessive disorders could be linked to regions of homozygosity (ROH) containing gene with point mutation which was inherited from related parents [2]. In this study, we showed additional two cases with autosomal recessive disorders which can be identified by high-resolution oligo-SNP array. The first was due to inheritance of ﻿alle﻿le﻿ with﻿ heterozygous deletion of different size from each carrier parent, which led to a homozygous deletion of *GLDC* gene (Fig. 4). The second was a homozygous deletion of the *CDK5RAP2* gene, inherited from closely related parents who carried the same heterozygous deletion (Additional file 3: Figure S2A). These two cases proved the efficacy of using high-resolution oligo-SNP array in the identification of extremely small homozygous pathogenic deletions.

## Conclusions

This study demonstrated 1): the incidence of recurrent small pathogenic deletions (< 1 Mb) was approximately 3 % (373/13,000) of all reported CNVs; 2): the small pathogenic deletions at 9p24.3 and 9q34.3 constituted 12 % of small pathogenic deletions from all the chromosomes, 3): 59 % of pathogenic deletions on chromosome 9 were due to small (<1 Mb) or relatively small (1–1.5 Mb) pathogenic deletions; 4): 81 % (46/57) of small pathogenic deletions were enriched at 9p24.3 and 9q34.3 regions involving the * DOCK8, KANK1* and *EHMT1* genes; 5): high-resolution oligo-SNP array was capable of identifying homozygous deletions as small as 25 kb in size.

## Methods

### Patients

Patients with a broad range of clinical indications including intellectual disability, developmental delay, multiple congenital anomalies, dysmorphic features and pervasive developmental disorders were referred to our laboratory for oligo-SNP array studies. The data for this study were compiled from de-identified results of 38,000 consecutive patient specimens referred to our laboratory for constitutional oligo-SNP array study from 2011 to 2015. The patients were majorly from general population in the United States, with < 5 % from Mexico and other countries.

### Oligonucleotide-single nucleotide polymorphism array analysis platforms and threshold setting

Oligo-SNP array analysis was performed on either Human SNP Array 6.0 (in 2011) or CytoScan® HD (2012–2015)(Affymetrix, Santa Clara, CA), using genomic DNA extracted from whole blood. The Human SNP Array 6.0 has 1.8 million genetic markers, including about 906,600 SNPs and 946,000 probes for the detection of CNVs. The CytoScan® HD has more than 2.67 million probes, including 1.9 million non-polymorphic copy number probes and 750,000 SNP probes. The overall resolutions are approximately 1.7 kb for Human SNP Array 6.0 and 1.15 kb for CytoScan® HD. For chromosome 9, the probes for Human SNP Array 6.0 covered: 9p(chr9:37,747-47,217,164) and 9q(chr9:65,596,318-141,091,382); for CytoScan HD®: 9p (chr9:192,129-40,784,142, chr9:43,400,082-44,900,526) and 9q (chr9:66,837,485-141,025,328). Genomic coordinates were based upon genome build 37/hg19 (2009). Hybridization, data extraction, and analysis were performed as per manufacturers’ protocols. The Affymetrix® Chromosome Analysis Suite (ChAS) Software version 2.0 was used for data analysis, review, and reporting. For genome-wide screening, thresholds were set at > 200 kb for gains and > 50 kb for losses. For cytogenetically relevant regions, thresholds were set at > 100 kb for gains and > 20 kb for losses. Benign CNVs that are documented in the database of genomic variations (http://dgv.tcag.ca/dgv/app/home?ref=GRCh37/hg19) and present in the general population were excluded from reporting.

## Additional files

Additional file 1: Figure S1. Profile of abnormal CNVs and VOUS in chromosome 9 from 531 cases. A total of 142 cases with pathogenic CNVs, and 389 cases with VOUS were identified. (JPG 52 kb)
Additional file 2: Table S1. List of the cases with small (<1 Mb) pathogenic copy number loss on chromosome 9 without involvement of other chromosomes (*N* = 57). **Table S2.** List of the remaining cases with copy number loss on chromosome 9 (*N* = 47). **Table S3.** Interpretation, references and ClinGen evaluation of haploinsufficiency score of selected cytogenetically relevant genes on chromosome 9. (DOCX 43 kb)
Additional file 3: Figure S2. A. The finding of multiple large regions of homozygosity (421 Mb) was due to closely parental relatedness. B. An approximately 74-kb homozygous deletion involved multiple exons of the *CDK5RAP2* gene in a region of homozygosity at 9q33.1-q34.11 (chr9:118,503,864-132,425,233), which caused autosomal recessive primary microcephaly-3 (OMIM #604804). (JPG 60 kb)

## Abbreviations

CMA: Chromosomal microarray analysis
CNV: Copy number variant
FISH: Fluorescence in situ hybridization
ISCN: International System of Cytogenetic Nomenclature
Oligo-SNP array: Oligonucleotide-single nucleotide polymorphism array
UPD: Uniparental disomy
VOUS: Variants of uncertain clinical significance
