# Supplementary figures and images for: Enrichment of small pathogenic deletions at chromosome 9p24.3 and 9q34.3 involving DOCK8, KANK1, EHMT1 genes identified by using high-resolution oligonucleotide-single nucleotide polymorphism array analysis
Source: Mol Cytogenet. 2016 Nov 15;9:82. doi: 10.1186/s13039-016-0291-3 (PMC5111223; doi:10.1186/s13039-016-0291-3)

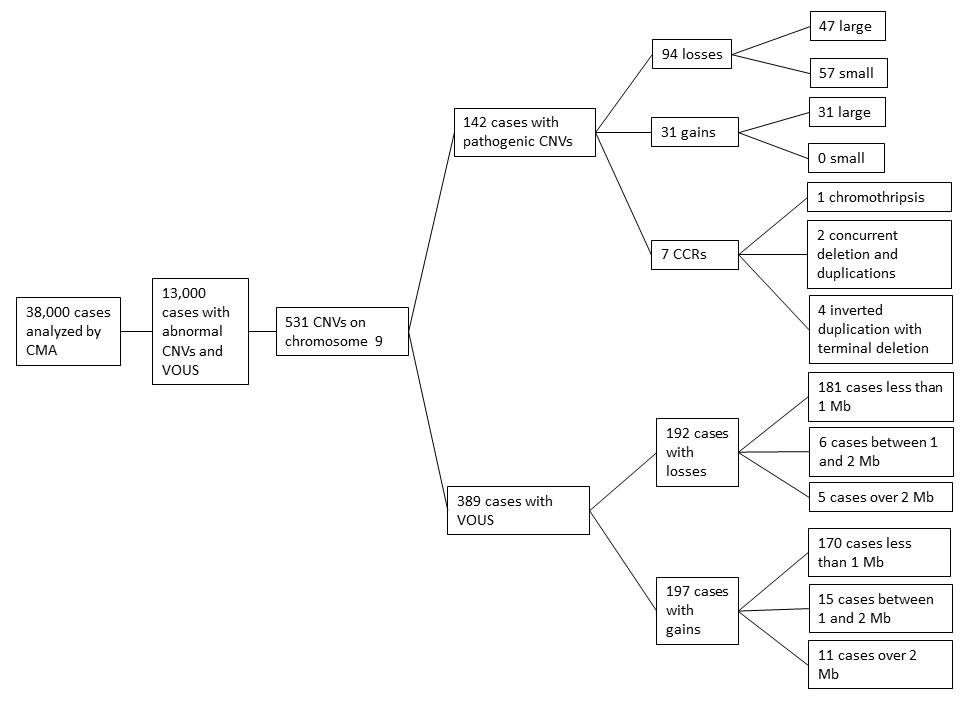

Supplement: Additional file 1: Figure S1. — Profile of abnormal CNVs and VOUS in chromosome 9 from 531 cases. A total of 142 cases with pathogenic CNVs, and 389 cases with VOUS were identified. (JPG 52 kb) [file 13039_2016_291_MOESM1_ESM.jpg]

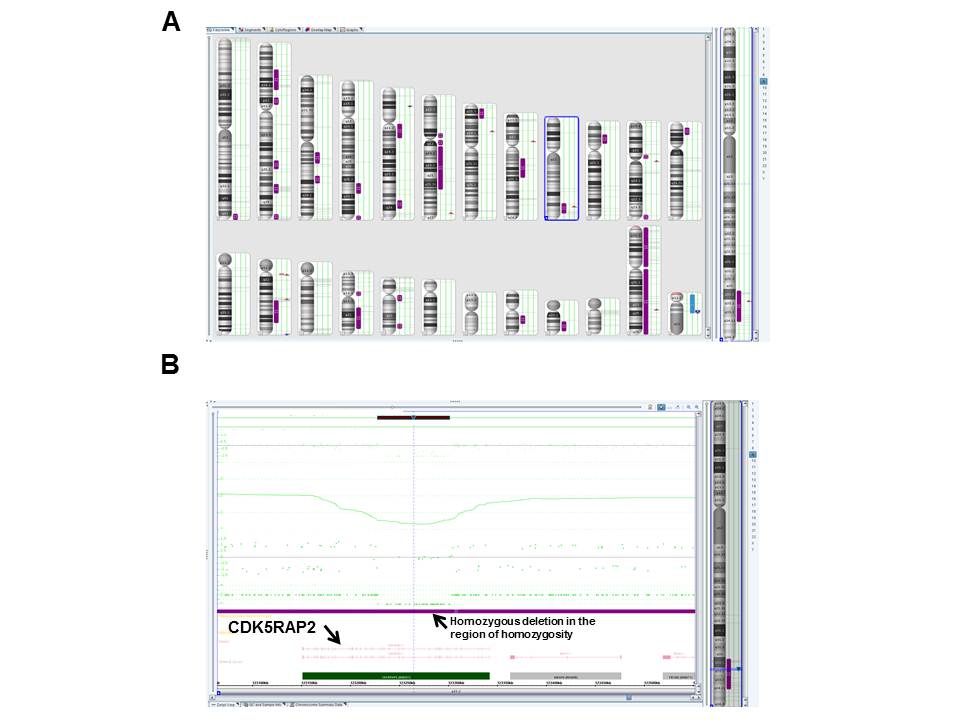

Supplement: Additional file 3: Figure S2. — A. The finding of multiple large regions of homozygosity (421 Mb) was due to closely parental relatedness. B. An approximately 74-kb homozygous deletion involved multiple exons of the CDK5RAP2 gene in a region of homozygosity at 9q33.1-q34.11 (chr9:118,503,864-132,425,233), which caused autosomal recessive primary microcephaly-3 (OMIM #604804). (JPG 60 kb) [file 13039_2016_291_MOESM3_ESM.jpg]
