# Supplementary material for: Enrichment of small pathogenic deletions at chromosome 9p24.3 and 9q34.3 involving DOCK8, KANK1, EHMT1 genes identified by using high-resolution oligonucleotide-single nucleotide polymorphism array analysis
Source: Mol Cytogenet. 2016 Nov 15;9:82. doi: 10.1186/s13039-016-0291-3 (PMC5111223; doi:10.1186/s13039-016-0291-3)
Supplement: Additional file 2: Table S1. — List of the cases with small (<1 Mb) pathogenic copy number loss on chromosome 9 without involvement of other chromosomes (N = 57). Table S2. List of the remaining cases with copy number loss on chromosome 9 (N = 47). Table S3. Interpretation, references and ClinGen evaluation of haploinsufficiency score of selected cytogenetically relevant genes on chromosome 9. (DOCX 43 kb) [file 13039_2016_291_MOESM2_ESM.docx]

**Additional file 2**

**TITLE:** Enrichment of small pathogenic deletions at chromosome 9p24.3 and 9q34.3 involving DOCK8, KANK1, EHMT1 genes identified by using high-resolution oligonucleotide-single nucleotide polymorphism array analysis

**Running Head:** Enrichment of small pathogenic deletions at 9p24.3 and 9q34.3

**AUTHORS**

Jia-Chi Wang^1^*, Loretta W. Mahon^1^, Leslie P. Ross^1^, Arturo Anguiano^1^, Renius Owen^1^, Fatih Z. Boyar^1^

**Abbreviations:**

CNV, copy number variant; ISCN, International System of Cytogenetic Nomenclature; VOUS, variants of uncertain clinical significance

Table S1. List of the cases with small (< 1 Mb) pathogenic copy number loss on chromosome 9 without involvement of other chromosomes (N = 57)

| Case | Start (bp) | End (bp) | Size (kb) | Disorder | Additional information | Brief clinical information |
| --- | --- | --- | --- | --- | --- | --- |
| 1 | 177044 | 229678 | 230 | 9p24.3 deletion | *DOCK8* deletion | 6-month-old male |
| 2 | 177044 | 329462 | 329 | 9p24.3 deletion | *DOCK8* deletion | 3-year-old male |
| 3 | 192128 | 429474 | 429 | 9p24.3 deletion | *DOCK8* deletion | 2-year-old male with developmental delay; behavior problem, speech delay; learning disability; autism spectrum disorder; ankyloglossia; midface hypoplasia |
| 4 | 203861 | 257999 | 258 | 9p24.3 deletion | *DOCK8* deletion | Male |
| 5 | 203861 | 258459 | 258 | 9p24.3 deletion | *DOCK8* deletion | Male |
| 6 | 203861 | 268590 | 269 | 9p24.3 deletion | *DOCK8* deletion | 2-year-old male |
| 7 | 203861 | 309108 | 309 | 9p24.3 deletion | *DOCK8* deletion | 5-year-old male |
| 8 | 203861 | 322786 | 323 | 9p24.3 deletion | *DOCK8* deletion | 6-year-old male |
| 9 | 203861 | 322786 | 323 | 9p24.3 deletion | *DOCK8* deletion | 2-year-old male |
| 10 | 203861 | 322786 | 323 | 9p24.3 deletion | *DOCK8* deletion | 4-year-old male |
| 11 | 203861 | 325832 | 326 | 9p24.3 deletion | *DOCK8* deletion | 7-year-old female |
| 12 | 203861 | 328178 | 328 | 9p24.3 deletion | *DOCK8* deletion | 4-year-old male with developmental delay, speech and motor delay, autism, macrocephalic, sensory hypersensitivity (auditory and tactile), feeding problems |
| 13 | 203861 | 360381 | 360 | 9p24.3 deletion | *DOCK8* deletion | 2-year-old male with macrocephaly, micrognathia, half-brother of case 14, maternally inherited |
| 14 | 203861 | 360381 | 360 | 9p24.3 deletion | *DOCK8* deletion | 7-year-old male; half-brother is case 13; maternally inherited |
| 15 | 203861 | 395276 | 395 | 9p24.3 deletion | *DOCK8* deletion | 15-year-old female with behavior problem; learning disability; dysmorphic features |
| 16 | 203861 | 402294 | 402 | 9p24.3 deletion | *DOCK8* deletion | 13-year-old male |
| 17 | 203861 | 430789 | 431 | 9p24.3 deletion | *DOCK8* deletion | 1-year-old male with developmental delay; brother (5 -year-old) with significant speech and language delay |
| 18 | 209753 | 399040 | 399 | 9p24.3 deletion | *DOCK8* deletion | 1-year-old male |
| 19 | 216123 | 399040 | 399 | 9p24.3 deletion | *DOCK8* deletion | 17-year-old male |
| 20 | 260680 | 436983 | 176 | 9p24.3 deletion | *DOCK8* deletion | 2-year-old male with mental retardation; brother of case 21 |
| 21 | 260680 | 436983 | 176 | 9p24.3 deletion | *DOCK8* deletion | 2-year-old male; brother of case 20 |
| 22 | 260680 | 436983 | 176 | 9p24.3 deletion | *DOCK8* deletion | 4-year-old male |
| 23 | 328094 | 424875 | 97 | 9p24.3 deletion | *DOCK8* deletion | 4-year-old male |
| 24 | 369485 | 455196 | 86 | 9p24.3 deletion | *DOCK8* deletion | 2-year-old male |
| 25 | 497478 | 1167288 | 670 | 9p24.3 deletion | *KANK1* deletion | 4-year-old female |
| 26 | 535205 | 718731 | 184 | 9p24.3 deletion | *KANK1* deletion | 9-year-old male |
| 27 | 540090 | 793275 | 253 | 9p24.3 deletion | *KANK1* deletion | 21-year-old male |
| 28 | 203861 | 506989 | 507 | 9p24.3 deletion | *DOCK8* and *KANK1* deletion | 6-year-old male with Chiari II malformation, obstructive hydrocephalus, seizures, dysphagia, developmental delay |
| 29 | 203861 | 676043 | 676 | 9p24.3 deletion | *DOCK8* and *KANK1* deletion | 1-year-old male |
| 30 | 424874 | 499997 | 75 | 9p24.3 deletion | *DOCK8* and *KANK1* deletion | 1-year-old male |
| 31 | 6581048 | 6655730 | 75 | *GLDC* heterozygous and homozygous deletion | Autosomal recessive glycine encephalopathy (nonketotic hyperglycinemia; OMIM #605899) inherited from bi-parental carrier of heterozygous deletion | Newborn female with meconium aspiration, abnormal reflexes, agenesis of the corpus callosum, hypotonia, possible seizures, elevated glycine levels |
| 32 | 14464111 | 15036880 | 573 | *FREM1* deletion | N/A | Newborn make |
| 33 | 14839293 | 15005043 | 166 | *FREM1* deletion | N/A | 39-year-old male; fetus with holoprosencephaly |
| 34 | 32259056 | 32595904 | 337 | *TOPORS* deletion | N/A | 17-year-old female with high-functioning autism, anxiety, encephalopathy |
| 35 | 119409559 | 119528928 | 119 | Disruption of *ASTN2* | N/A | 8-year-old male |
| 36 | 119474699 | 119552869 | 78 | Disruption of *ASTN2* | N/A | 12-year-old female with autism, strabismus |
| 37 | 119487225 | 119619937 | 133 | Disruption of *ASTN2* | Maternally inherited | 5-year-old female with speech delay, learning disability, height 95%tile, weight >99%tile, bed wetting, left foot 2/3 syndactyly, increase in weight gain |
| 38 | 119525072 | 119795696 | 271 | Disruption of *ASTN2* | N/A | 1-year-old male with gross motor and language delays, low muscle tone, prominent epicanthal folds, protuberant tongue, wide nuchal base, elevated CPK at 185 |
| 39 | 123227720 | 123301396 | 74 | Homozygous loss of *CDK5RAP2* | arr[hg19] 1q43q44(241,180,996-249,198,692)x2 hmz, 2p12p11.2(75,414,091-85,331,686)x2 hmz, 2p22.2p14(36,896,843-65,106,265)x2 hmz, 2q24.2q31.1(161,844,819-173,284,088)x2 hmz, 2q32.3q33.3(194,009,785-206,464,032)x2 hmz, 2q37.1q37.3(235,519,241-242,775,910)x2 hmz, 3q13.11q13.33(105,338,151-120,674,779)x2 hmz, 3q22.3q24(137,573,306-147,951,851)x2 hmz, 4q31.1q31.3(141,053,847-155,150,556)x2 hmz, 4q35.1q35.2(185,436,254-190,921,709)x2 hmz, 5q11.1q13.2(49,560,858-68,826,246)x2 hmz, 5q33.2q34(154,207,967-165,318,588)x2 hmz, 6p12.2p11.1(52,565,210-58,741,497)x2 hmz, 6q11.1q12(61,968,745-68,819,976)x2 hmz, 6q13q22.33(70,579,103-129,391,343)x2 hmz, 7p22.1p21.1(6,761,800-20,302,184)x2 hmz, 8q12.2q21.3(62,004,771-88,528,370)x2 hmz, 9q33.1q34.11(118,503,864-132,425,233)x2 hmz, 10p12.33p11.23(18,481,043-30,853,414)x2 hmz, 11p11.2p11.12(46,364,242-51,563,636)x2 hmz, 11q24.3q25(128,163,456-134,217,987)x2 hmz, 12p13.31p12.3(8,116,895-17,761,222)x2 hmz, 14q23.1q32.11(58,495,376-90,580,987)x2 hmz, 16p11.2p11.1(29,289,976-35,220,544)x2 hmz, 16q12.1q23.3(51,913,846-81,992,906)x2 hmz, 17q11.1q12(25,309,336-33,539,825)x2 hmz, 17q24.2q25.1(65,657,495-72,201,796)x2 hmz, 20q11.23q13.13(35,500,057-47,471,218)x2 hmz, 21q21.3q22.3(29,284,969-43,086,816)x2 hmz  A total of 421 Mb ROH | 1-year-old male |
| 40 | 130230741 | 130822806 | 592 | *STXBP1* deletion | N/A | 2-year-old female |
| 41 | 137443423 | 137813047 | 370 | *COL5A1* deletion | N/A | 5-year-old female |
| 42 | 140170355 | 140892258 | 722 | Kleefstra syndrome | Deletion of 9 OMIM genes including *EHMT1* | 2-year-old female with global developmental delay, diaphragmatic hernia |
| 43 | 140230197 | 140652571 | 422 | Kleefstra syndrome | Deletion of 9 OMIM genes including *EHMT1* | 13-year-old female with autism spectrum disorder |
| 44 | 140230197 | 141020389 | 790 | Kleefstra syndrome | Deletion of 8 OMIM genes including *EHMT1* | 2-year-old male |
| 45 | 140255704 | 141020389 | 765 | Kleefstra syndrome | Deletion of 8 OMIM genes including *EHMT1* | 16-year-old female |
| 46 | 140267468 | 141020389 | 753 | Kleefstra syndrome | Deletion of 8 OMIM genes including *EHMT1* | 9-year-old female |
| 47 | 140276341 | 141020389 | 744 | Kleefstra syndrome | Deletion of 8 OMIM genes including *EHMT1* | 14-year-old female |
| 48 | 140390614 | 141064741 | 674 | Kleefstra syndrome | Deletion of 6 OMIM genes including *EHMT1* | 20-year-old female |
| 49 | 140390614 | 141091382 | 701 | Kleefstra syndrome | Deletion of 6 OMIM genes including *EHMT1* | 1-year-old male |
| 50 | 140475648 | 140612644 | 137 | Kleefstra syndrome | Involving *EHMT1* only | 11-year-old male |
| 51 | 140493726 | 140659055 | 165 | Kleefstra syndrome | Involving *EHMT1* only | Newborn male |
| 52 | 140650471 | 140689373 | 39 | Kleefstra syndrome | Involving *EHMT1* only | 1-year-old female with developmental delay, speech and motor delay, white matter changes on MRI, hypotonia |
| 53 | 140651785 | 141020389 | 369 | Kleefstra syndrome | EHMT1 and CACNA1B deletion | 17-year-old male |
| 54 | 140667619 | 140689934 | 22 | Kleefstra syndrome | Involving *EHMT1* only | 32-year-old female with Intellectual disability |
| 55 | 140691671 | 140857772 | 166 | Kleefstra syndrome | Involving *EHMT1* and *CACNA1B* | 13-year-old male with de novo deletion (on the paternal allele) |
| 56 | 140694541 | 140734178 | 40 | Kleefstra syndrome | Involving *EHMT1* only | 5-year-old female |
| 57 | 140694541 | 140859507 | 165 | Kleefstra syndrome | Involving *EHMT1* and *CACNA1B* | 26-year-old male with mental retardation, developmental delay, speech delay, motor delay, learning disability, seizure, autism spectrum disorder, asymmetry of temporal lobe, localized polymicrogyria, loping gait, scoliosis |

Table S2. List of the remaining cases with copy number loss on chromosome 9 (N = 47)

| Case | Disorder | Start (bp) | End (bp) | Size (kb) | Diagnosis and karyotyping | Brief clinical information |
| --- | --- | --- | --- | --- | --- | --- |
| 1 | Interstitial deletion | 1634617 | 5041098 | 3406 | 9p deletion syndrome (OMIM #158170) | Hypotonia, developmental delay, prominent forehead, flat profile, bulbous nose, low set ears, single palmar creases, fleshy hands, mild short 4th metacarpals, pes cavus, wide-spaced nipples, positive femoral pulses |
| 2 | Interstitial deletion | 3012371 | 14585866 | 11573 | 9p deletion syndrome (OMIM #158170) | N/A |
| 3 | Interstitial deletion | 7748384 | 12230701 | 4482 | 9p deletion syndrome (OMIM #158170) | Intellectual disability (IQ: mid 70), developmental delay, behavior problem |
| 4 | Interstitial deletion | 7748384 | 12230701 | 4482 | 9p deletion syndrome (OMIM #158170) | Intellectual disability, developmental delay, learning disability, autism spectrum disorder, long thin face, large ears |
| 5 | Interstitial deletion | 30333038 | 32470575 | 2138 | Insertion of 9p to 1p; 46,XY,ins(1;9)(p32;p24p21),del(7)(q21.11q21.12) | N/A |
| 6 | Interstitial deletion | 68240211 | 75764536 | 7524 | 46,XX,del(9)(q12.q21.2) | Mild intellectual disability, developmental delay, speech delay, flat profile, bulbous nose, long fingers, scoliosis, Hashimoto's hypothyroidism |
| 7 | Interstitial deletion | 72702311 | 75951276 | 3249 | 9q interstitial deletion | Developmental delay, motor delay, learning disability, central auditory processing disorder, ADHD, one febrile seizure, borderline short stature, low weight, hypotonia, FH-mom with bifid uvula, history of hand cramping, B12 deficiency and increased MMA |
| 8 | Interstitial deletion | 90423565 | 107260599 | 16837 | Gorlin syndrome with *PTCH1* deletion | Perinatal depression s/p body cooling |
| 9 | Interstitial deletion | 90438907 | 102250295 | 11811 | Gorlin syndrome with *PTCH1* deletion | Macrocephaly, micrognathia |
| 10 | Interstitial deletion | 97659666 | 98707607 | 1048 | Gorlin syndrome with *PTCH1* deletion | Developmental delay |
| 11 | Interstitial deletion | 104467205 | 110486599 | 6019 | 9q interstitial deletion | N/A |
| 12 | Interstitial deletion | 107220339 | 109806237 | 2586 | 9q interstitial deletion | N/A |
| 13 | Interstitial deletion | 108657101 | 115356416 | 6699 | 9q interstitial deletion | N/A |
| 14 | Interstitial deletion | 129616292 | 132305322 | 2689 | 9q interstitial deletion, involving *STXBP1* gene associated with Epileptic encephalopathy, early infantile, 4 | N/A |
| 15 | Interstitial deletion | 130047266 | 131396223 | 1349 | 9q interstitial deletion, involving *STXBP1* gene associated with Epileptic encephalopathy, early infantile, 4 | N/A |
| 16 | Interstitial deletion | 131569659 | 133262920 | 1693 | 9q interstitial deletion | N/A |
| 17 | Interstitial deletion | 132793003 | 134594035 | 1801 | 9q interstitial deletion | N/A |
| 18 | Interstitial deletion | 137231719 | 139104812 | 1873 | Ehlers-Danlos syndrome, involving *COL5A1* gene | N/A |
| 19 | Terminal deletion of 9p | 37746 | 4005463 | 4005 | 9p deletion syndrome (OMIM #158170), de novo | Developmental delay |
| 20 | Terminal deletion of 9p | 203861 | 2528094 | 2528 | 9p deletion syndrome (OMIM #158170) | N/A |
| 21 | Terminal deletion of 9p | 203861 | 3720298 | 3720 | 9p deletion syndrome (OMIM #158170) | N/A |
| 22 | Terminal deletion of 9p | 203861 | 5844828 | 5845 | 9p deletion syndrome (OMIM #158170) | N/A |
| 23 | Terminal deletion of 9p | 203861 | 7566748 | 7567 | 9p deletion syndrome (OMIM #158170) | N/A |
| 24 | Terminal deletion of 9p | 203861 | 10542639 | 10543 | 9p deletion syndrome (OMIM #158170) | N/A |
| 25 | Terminal deletion of 9p | 203861 | 11826196 | 11826 | 9p deletion syndrome (OMIM #158170) | N/A |
| 26 | Terminal deletion of 9p | 203861 | 13592591 | 13593 | 9p deletion syndrome (OMIM #158170) | N/A |
| 27 | Terminal deletion of 9p | 402293 | 6008933 | 6009 | 9p deletion syndrome (OMIM #158170) | N/A |
| 28 | Terminal deletion of 9q | 138959881 | 141020389 | 2061 | 9q34.3 deletion syndrome (OMIM #610253) | N/A |
| 29 | Terminal deletion of 9q | 139217670 | 141020389 | 1803 | 9q34.3 deletion syndrome (OMIM #610253) | N/A |
| 30 | Terminal deletion of 9q | 139257286 | 141091382 | 1834 | 9q34.3 deletion syndrome (OMIM #610253) | N/A |
| 31 | Terminal deletion of 9q | 139802129 | 141020389 | 1218 | 9q34.3 deletion syndrome (OMIM #610253) | N/A |
| 32 | Terminal deletion of 9q | 139808638 | 141076929 | 1268 | 9q34.3 deletion syndrome (OMIM #610253) | N/A |
| 33 | Unbalanced translocation | 37746 | 2809548 | 2810 | t(9;12)(p24.2;p11.1) | N/A |
| 34 | Unbalanced translocation | 37746 | 8236236 | 8236 | t(4;9)(p15.1;p24.1) | Developmental delay; dysmorphic features; midline hemangioma |
| 35 | Unbalanced translocation | 203861 | 1004821 | 1005 | t(9;13)(p24.3;q31.1) | N/A |
| 36 | Unbalanced translocation | 203861 | 4370539 | 4371 | t(9;13)(p24.2;q31.1) | Extra digit, hearing deficit |
| 37 | Unbalanced translocation | 203861 | 5041098 | 5041 | t(1;9)(p36.31;p24.1) | N/A |
| 38 | Unbalanced translocation | 203861 | 5374687 | 5375 | t(9;16)(p24.1;q22.2) | Developmental delay, microcephaly (2SD below 2%), behavior problem, speech delay, heart murmur, epicanthal folds, thin upper lip, flat philtrum, foster care |
| 39 | Unbalanced translocation | 203861 | 7860213 | 7860 | t(9;10)(p24.1;p12.1) | Intellectual disability, developmental delay, microcephaly, hypotonia |
| 40 | Unbalanced translocation | 203861 | 12117273 | 12117 | t(9;11)(p23;q23.2) | N/A |
| 41 | Unbalanced translocation | 203861 | 12899064 | 12899 | t(6;9)(p22.3;p23) | N/A |
| 42 | Unbalanced translocation | 203861 | 13049945 | 13050 | t(9;18)(p23;q22.3) | Developmental delay; speech delay; learning disability; cupped ears; high arched palate; Gower sign; abnormal gait; flat feet; maternal male cousin speech/learning disability |
| 43 | Unbalanced translocation | 203861 | 13064793 | 13065 | (7;9)(p21.2;p23)mat | N/A |
| 44 | Unbalanced translocation | 203861 | 17106922 | 17107 | t(7;9)(p22.1;p22.2) | N/A |
| 45 | Unbalanced translocation | 140018931 | 141020389 | 1001 | t(9;21)(q34.3;q22.3) | Developmental delay, speech delay, prominent pituitary cysts, small corpus callosum, small brain stem, flat midface, depressed nasal bridge, downslanting palpebral fissures, synophrys |
| 46 | Unbalanced translocation | 140034826 | 141020389 | 986 | t(9;10)(q34.3;q26.13) | N/A |
| 47 | Unbalanced translocation | 140140868 | 141020389 | 880 | t(9;11)(q34.3;p15.4) | N/A |

Table S3. Interpretation, references and ClinGen evaluation of haploinsufficiency score of selected cytogenetically relevant genes on chromosome 9

| Deletion | Interpretation and references | ClinGen (Date) |
| --- | --- | --- |
| *DOCK8* deletion | Disruption of the *DOCK8* gene, by deletion or translocation, has been reported to be causative of the autosomal dominant mental retardation-2 syndrome (OMIM #614113). Other clinical features may include absence of speech, mild dysmorphic features, a history of seizures, and stooped posture (Griggs et al., Genomics 91: 195-202, 2008; PMID: 18060736). | Haploinsufficiency Score: gene associated with autosomal recessive phenotype (ClinGen did not mention the autosomal dominant mental retardation-2 syndrome); Last evaluation 2016-08-22 |
| *KANK1* deletion | The deletion of exons of the *KANK1* gene has been reported to cause parent-of-origin-dependent inheritance of familial cerebral palsy when deleted (Lerer, et al., Hum Mol Genet. 2005 Dec 15;14(24):3911-20; PMID: 16301218). However, a paternally inherited deletion in a recently reported proband's unaffected sibling did not support maternal imprinting raising consideration of further complexity of the *KANK1* locus, including variable expressivity, incomplete penetrance, and the additive effects of additional genomic variants or the potential benign nature of the variation (Vanzo, et al., Eur J Med Genet. 2013 May;56(5):256-9; PMID: 23454270). | Haploinsufficiency Score: 0, (Triplosensitivity Score: 0); Last evaluation 2016-04-28 |
| *EHMT1* deletion | The deletion of the *EHMT1* gene is expected to cause phenotypic and/or developmental abnormalities. Submicroscopic subtelomeric deletions of chromosome 9q are associated with 9q34.3 deletion syndrome (OMIM #610253). Common features in patients with 9q subtelomeric deletion syndrome are severe mental retardation, hypotonia, brachy(micro)cephaly, epileptic seizures, flat face with hypertelorism, synophrys, anteverted nares, everted lower lip, carp mouth with macroglossia, and heart defects (Harada, et al., J. Hum. Genet. 49: 440-444, 2004. PMID: 15258833; Iwakoshi, et al., Am. J. Med. Genet. 126A: 278-283, 2004. PMID: 15054842). | Haploinsufficiency Score: 3, (Triplosensitivity, score: 1); Last evaluation 2012-07-06 |
| *PTCH1* deletion | Haploinsufficiency of *PTCH1*, due to deletion or loss of function mutations, has been linked to nevoid basal cell carcinoma syndrome (Gorlin syndrome OMIM #109400). Gorlin syndrome is an autosomal dominant disorder that can be manifested with multiple basal cell carcinomas (BCCs), cysts of the jaws, hyperkeratosis of palms and soles, skeletal abnormalities (bifid ribs /hemivertebrae), intracranial ectopic calcifications, facial dysmorphism, and increased risk for medulloblastoma (Lo Muzio, Orphanet J Rare Dis. 2008 Nov 25;3:32; PMID: 19032739 ). Mutations of *PTCH1* gene have also been linked to holoprosencephaly-7 anomaly (OMIM #610828) manifested by mental retardation and craniofacial malformation (Ming etal, Hum Genet. 2002 Apr;110(4):297-301; PMID: 11941477). The gene encoding for Fanconi's anemia complementation group C (FANCC) is one of the genes implicated in this genetically heterogenous autosomal recessive disease characterized mainly by bone marrow failure (Lo Ten Foe et al, Eur J Hum Genet. 1997 May-Jun;5(3):137-48; PMID: 9272737). | Haploinsufficiency Score: 3 (Triplosensitivity, score: 1); Last evolution 2011-12-01 |
| *ASTN2/TRIM32* deletion | Studies suggest the deletion at 9q33.1, including *TRIM32* and several exons of *ASTN2*, may be pathogenic. Compound heterozygous or homozygous *TRIM32* mutations alone can cause either of two autosomal recessive conditions, Bardet-Biedl syndrome 11 (OMIM #209900) and limb-girdle muscular dystrophy, type 2H (OMIM #254110). More notably, disruption of *ASTN2* is found to be a risk factor for males for autism spectrum disorders, ADHD and other neurodevelopmental phenotypes (Nectoux, et al., Eur J Hum Genet. 2014 Oct 29. PMID: 25351777. Lionel, et al., Hum Mol Genet. 2014 May 15;23(10):2752-68. PMID: 24381304). | Awaiting review, last review 2015-10-05 |
| *FREM1* deletion | This loss at 9p partially involves the *FREM1* gene (OMIM 608944). Heterozygous mutations and haploinsufficiency of *FREM1* is associated with trigonocephaly 2 (OMIM #614485). Homozygous and compound heterozygous mutations within the gene are associated with bifid nose with or without anorectal and renal anomalies (OMIM #608980) and Manitoba oculotrichoanal syndrome (OMIM #248450). | Haploinsufficiency Score: 0; Last evaluation 2013-06-06 |
| *STXBP1* deletion | The loss of syntaxin binding protein 1 (*STXBP1*; OMIM 602926 ) has been implicated in epileptic encephalopathy, early infantile, 4 (OMIM 612164; Saitsu, H. et al. Nature Genet. 40: 782-788, 2008; PMID: 18469812). | Haploinsufficiency Score: 3, (Triplosensitivity, score: 0); Last evaluation 2013-06-20 |
| *COL5A1* deletion | *COL5A1* haploinsufficiency is a common molecular mechanism underlying the classical form of Ehlers-Danlos syndromes (EDS, OMIM #130000 and #130010; Wenstrup, et al., Am J Hum Genet. 2000 Jun;66(6):1766-76; PMID: 10777716). EDS are a group of heritable connective tissue disorders that share the common features of skin hyperextensibility, articular hypermobility, and tissue fragility. The main features of classic Ehlers-Danlos syndrome (EDS I and EDS II) are loose-jointedness and fragile, bruisable skin that heals with peculiar 'cigarette-paper' scars. Additional genes within the deleted region may also contribute to a more severe phenotype. | Awaiting review, last review 2015-10-05 |
| *GLDC deletion* | Homozygous and compound heterozygous mutations or deletions of the *GLDC* gene (OMIM 238300) are associated with the glycine encephalopathy (nonketotic hyperglycinemia; OMIM #605899). If the patient is or has been on valproic acid therapy, it is recommended to carefully replace it with another anticonvulsion medication since valproic acid may interfere with the glycine cleavage system. | Haploinsufficiency Score: gene associated with autosomal recessive phenotype (Triplosensitivity Score: 0); Last evaluation 2016-06-10 |
| *TOPORS* deletion | This loss contains the *TOPORS* gene which is associated with autosomal dominant retinitis pigmentosa 31 (OMIM #609923). Studies of patients with two different frameshift mutations suggest that haploinsufficiency of *TOPORS* may be the molecular mechanism for the disease (Chakarova, et al., Am. J. Hum. Genet. 81: 1098-1103, 2007; PMID: 17924349) although one study of control individuals reported two cases with deletions over this gene (Matsuzaki, et al., Genome Biol. 2009;10(11):R125; PMID: 19900272). | Awaiting review, last evaluation 2015-10-05 |
| Homozygous deletion of *CDK5RAP2* | This assay exhibited an approximately 74 kb homozygous loss within 9q33.2 in a male. The variation is within one of multiple regions of allelic homozygosity that were also detected. The homozygous deletion involves several exons of the *CDK5RAP2* gene and is expected to cause phenotypic and/or developmental abnormalities. Primary microcephaly-3 (OMIM #604804) is caused by homozygous or compound heterozygous mutation in the *CDK5RAP2.* | Haploinsufficiency Score: gene associated with autosomal recessive phenotype (Triplosensitivity Score: Not yet evaluated); Last evaluation 2016-08-22 |
